# Supplementary material for: It’s how you say it: Systematic A/B testing of digital messaging cut hospital no-show rates
Source: PLoS One. 2020 Jun 23;15(6):e0234817. doi: 10.1371/journal.pone.0234817 (PMC7310733; doi:10.1371/journal.pone.0234817)
Supplement: S1 Table — (DOCX) [file pone.0234817.s001.docx]

**Supplement 1. Codes used for variable definitions.**

| **Input Variable** | **Relevant Extraction Codes ^†,‡^** |
| --- | --- |
| **Cardiovascular disease** | ICD-9 codes: 410%; 411%; 412%; 413%; 414.[0189]%; 43[14568]%; 437.[01]; 437  ICPC codes: K75; K76; K90  Relevant CHS chronic registry codes |
| **Diabetes ( type 1 and type 2)** | Step 1: All diabetic members were extracted using ICD-9 code of 250% or relevant CHS chronic registry codes. Step 2: Number of specific diagnosis phrases or codes indicating type 1 diabetes (any ICD-9 code of 250.1% or other codes with a fifth digit of 1 or 3) were compared to the number of diagnosis phrases or codes indicating type 2 diabetes (codes with a fifth digit of 0 or 2) were compared for each individual. Step 3: Purchase of medication specific for type 2 diabetes were extracted (ATC3 code of A10B given per os, excluding ATC5 codes of A10BA02, A10BX12, or A10BX09). Step 4: Any individual extracted in step 1 who had more specific type 1 diagnoses than specific type 2 diagnoses (step 2) and did not purchase type 2 medication (step 3), was considered to have type 1 diabetes. Step 5: Any individual extracted in step 1 who was not coded as having type 1 diagnosis in step 4, was considered to have type 2 diabetes. |
| **Chronic Kidney Disease** | ICD-9 codes: 585%; 58[257]%; 250.4%; V42.0 and last eGFR prior to index date less than 60 ml/min/1.73m2 |
| **Celiac** | 579% |
| **Inflammatory bowel disease** | 555%; 556% |

^†^ All diagnoses made in community and hospital settings were further validated using free text phrases of the doctor's notes (which only exists for these records), as well as from the CHS chronic disease registry.

^‡^ There are two shorthand methods used for coding: 1) Codes marked with %: the % is used to extract all sub-codes of the higher-level code, with the % representing a place in the code that can be substituted by any number of characters (e.g., 820% will be used to extract 820.1…820.9); and 2) Codes marked with [ ]: The square brackets represent a single digit in the code that can be substituted by any one of the included digits (e.g., the code 733.[01] is used to extract both codes 733.0 and 733.1).
